# Migraine, associated factors, and functional disability in medical students at a peruvian university during the COVID-19 pandemic: An analytical cross-sectional study

**DOI:** 10.1016/j.heliyon.2023.e18108

**Published:** 2023-07-08

**Authors:** Annabell Zevallos-Vásquez, Kiana Azucena Pajuelo-Salazar, J. Jhonnel Alarco

**Affiliations:** School of Medicine, Faculty of Health Sciences, Universidad Científica Del Sur, Lima, Peru

**Keywords:** Migraine disorders, Medical students, Disability evaluation, COVID-19

## Abstract

**Background:**

It is unknown whether the confinement caused by the coronavirus disease 2019 (COVID-19) pandemic may influence migraine triggers. This study aimed to determine the frequency of migraine and their associated factors in medical students at a Peruvian university. The characteristics of migraine episodes and degree of functional disability caused by migraine were also evaluated.

**Methods:**

An analytical cross-sectional study was conducted on medical students of a Peruvian university. The suspected migraine was determined with the Migraine Screen Questionnaire. Sociodemographic, confounding, and COVID-19 pandemic–related variables were included as possible associated factors. Poisson regression models were used, and crude and adjusted prevalence ratios with 95% confidence intervals were estimated.

**Results:**

The participants comprised 327 students, and 30.3% (n = 99) had suspected migraine. Sex, clustered semesters, having family members with migraine and COVID-19 risk factors, and hours in front of the computer were associated with a higher probability of migraine. However, having more hours of sleep per day was associated with a lower probability. Moreover, most students with migraine had a severe functional disability (41.4%).

**Conclusions:**

The high frequency of migraine in medical students could be due to COVID-19-related quarantine. Certain pandemic-related factors increase the probability of having migraine. A high level of disability should promote timely diagnosis and follow-up in affected students.

## Abbreviations

COVID-19: Coronavirus disease 2019
MS-Q: Migraine screen questionnaire
MIDAS: Migraine disability assessment questionnaire
PR: Prevalence ratio
VIF: Variance inflation factor
HBP: High Blood Pressure

## 1 Introduction

According to the World Health Organization, migraine is the sixth cause of years lost due to disability, impaired quality of life, and altered family and social relationships [1]. The prevalence of migraine in the United States is 12%, with a frequency of 18% in women and 6% in men [2]. In Latin America, the prevalence of migraine varies in each country, with higher proportions in Brazil (29.5%) and lower in Colombia (7.1%) and Peru (5.3%) [3].

Some studies have analyzed migraine in Peru's high-altitude populations. A prevalence of 5.3% was recorded in Cuzco [4] and 12.4% in Cerro de Pasco [5]. In hospital studies, a prevalence of 29.2% was registered in pregnant women [6] and 21.2% in postpartum adolescent mothers [7].

The coronavirus disease 2019 (COVID-19) pandemic has significantly impacted the academic training of students. The closure of universities has given way to virtual classes [8], which, despite being well accepted [9], have caused high levels of stress [10] and anxiety [11] in medical students.

It is not known whether the confinement caused by the pandemic can influence migraine triggers. The results are still conflicting. One study of patients with migraine identified an increase in migraine frequency in more than 50% of participants, increasing both the severity of these episodes and use of over-the-counter analgesics [12]. Another study found a reduction in the length of migraine episodes and clinical improvement in 47.1% of the patients, revealing that confinement positively affected migraine occurrence [13]. There is abundant evidence supporting the fact that confinement has affected the lifestyles of people with migraine, altering sleep quality and reducing levels of physical activity [14]. It has also been described that new work habits have favored rest and self-care, decreasing the intensity of migraine [15].

Therefore, this study aimed to determine the frequency of migraine episodes and their associated factors in medical students from a Peruvian university during the COVID-19 pandemic. Likewise, the characteristics of migraine episodes and the degree of functional disability caused by migraine were evaluated.

## 2 Materials and methods

### 2.1 Design and population

In 2021, an analytical cross-sectional study was conducted on medical students at the Universidad Científica del Sur (UCSUR), which is a private university with five faculties and 24 university programs; by 2021, it enrolled 14,178 students as undergraduates. The Human Medicine program enrolled 3700 students, with 2432 (65.1%) female and 1268 (34.9%) male students (https://bit.ly/ucsur2021-1).

### 2.2 Selection criteria

The sample comprised students enrolled in the 2021-1 academic year of the 3rd–12th semesters of the Human Medicine program who were >18 years old and accepted the virtual informed consent form. Students who answered the virtual questionnaire incompletely or inconsistently were excluded.

### 2.3 Sample and sampling

We considered 1980 students (semesters 3–12) according to the records of students enrolled in the 2020-2 academic year (https://bit.ly/ucsur2020-2). A maximum migraine prevalence of 50% was assumed as there were no similar studies on medical students during the pandemic. The calculated sample size was 322 students with a 95% confidence interval (CI), two-tailed, with a precision of 0.05 when the actual proportion approaches 50%. PASS version 15.0.5 was used for this calculation.

A non-probabilistic sampling by quotas according to academic semester and sex was conducted. The sample was divided into 10 quotas according to the academic semester, and each semester was divided into 65% female and 35% male students, according to the overall proportion of UCSUR medical students in 2020 (https://bit.ly/ucsur2020-2).

### 2.4 Recruitment

A virtual questionnaire that included all the variables of interest was developed. It was disseminated through the official social networks of the UCSUR, which are only used by students. To ensure adequate participation and in order to obtain a sufficient sample of students, the virtual questionnaire was disseminated through five stages.

• Stage 1: The virtual questionnaire was published every three days on the official medical students' Facebook page. This stage lasted a month. Since the sample was still incomplete after this stage, we moved on to stage 2.
• Stage 2: The virtual questionnaire was sent to medical students via e-mail. This stage also lasted a month. And since the sample was still incomplete after this stage, we moved on to stage 3.
• Stage 3: The delegates of each course were asked to disseminate the virtual questionnaire in their WhatsApp groups every three days. This stage lasted a month as well. The sample was still incomplete after this stage, so we moved on to stage 4.
• Stage 4: After identifying the semesters with the lowest response rate, Stages 2 and 3 were repeated only for the students in those semesters. This stage also lasted a month. Since the sample was still incomplete after this stage, we moved on to stage 5.
• Stage 5: For the semesters with the lowest response rate, the authors asked their acquaintances to disseminate the questionnaire among their classmates (snowball technique). This stage lasted one month.

### 2.5 Survey administration

Data were collected through a virtual questionnaire elaborated in Google Forms (https://docs.google.com/forms/). This strategy was ideal in this particular public health context in which students had to maintain social distancing. No financial or other incentive was provided for participating. The questionnaire was prepared with 40 questions (Supplementary Material). The following categories were not included: “Other,” “don't know,” or “no response”. Filling out the questionnaire took approximately 7 min. The questionnaire was available online from June 15, 2021, to November 26, 2021.

### 2.6 Variables

Migraine was measured with the migraine screen questionnaire (MS-Q) developed by Lainez et al. in 2005 [16], which has five questions related to headache frequency, characteristics and the presence or absence of migraine-related symptoms. Each negative response (no) scored 0, and each positive response (yes) scored 1. A score ≥4 points indicated suspicion of migraine, whereas a score <4 points indicated no suspicion. This instrument has a sensitivity of 93% and specificity of 81% and has been applied to students in Peru [17,18].

Functional disability was assessed with the migraine disability assessment scale (MIDAS), which measures disability related to days lost due to migraine. The instrument has five questions on work, domestic, and socio-family situations. The scoring system is divided into grade I, no disability or low disability (0–5 points); grade II, mild disability (6–10 points); grade III, moderate disability (10–20 points); and grade IV, severe disability (>21 points). Stewart et al. published this scale in 1999 [19]; since then, it has been widely used in several studies worldwide. It also has a validated version in Spanish [20]. In Peru, Adeney et al. [6] used it on pregnant women attending a prenatal care clinic in Lima, and Ayala [18] used it on medical students.

We included sociodemographic variables such as sex (male/female), age (years), grouped semesters (basic [semesters 3–6]; clinical [semesters 7–12]), and family income (<6000 soles and >6000 soles).

We also included possible confounding variables such as body mass index (thin/normal or overweight/obese) according to the self-reported weight (kg) and height (cm), family members with migraine (no, first degree, or second degree), chronic diseases (no/yes), insomnia during the pandemic (no/yes), exercise during the pandemic (no/yes), hours in front of the computer, hours of sleep per day, use of prescription glasses (no/yes), consumption of food high in fats (once a week or two or more times a week), coffee consumption (no/yes), alcohol consumption (no/yes), tobacco use (no/yes), and consumption of stimulant beverages (no/yes).

We also included pandemic-related variables: do you have or have you had COVID-19? (no/yes), do you have or have you had a family member with COVID-19? (no/yes), do you have close family members with risk factors for getting sick with COVID-19 (HBP [High Blood Pressure], diabetes, obesity, older adult, etc.)? (no/yes), and are you afraid of getting infected with COVID-19? (not afraid or little/regular/very afraid).

Students who had suspected migraine described the frequency, duration and intensity, symptoms, and treatment. The functional disability caused by migraine was also determined.

### 2.7 Statistical processing and analysis

The survey results were downloaded from the Google Forms platform in a. csv file and imported into Stata® version 16.0 for Windows. The Shapiro–Wilk test was used to evaluate the distribution of the numerical variables (age, hours of sleep per day, and hours in front of the computer). All presented normal distribution (p > 0.05), so parametric statistics were used.

During the univariate analysis, we calculated percentages and frequencies for categorical variables and the mean with standard deviation (SD) for numerical variables. During the bivariate analysis, differences were assessed according to the presence or absence of the suspicion of migraine. The chi-square test was used for categorical variables, and Student's t-test for numerical variables. Two models were created for the multivariate analysis, namely, a crude model and an adjusted model. The variables associated (p < 0.05) in the crude model were included in the adjusted model. Prevalence ratios with their 95%CI were estimated by using a generalized linear model of Poisson regression with logarithmic link and robust variance. A value of p < 0.05 was considered to be significant. Moreover, the presence of multicollinearity in the adjusted model was evaluated by calculating the variance inflation factor (VIF). A VIF ≥10 indicated multicollinearity.

## 3 Results

A total of 332 surveys were collected, of which 5 were excluded (4 with missing data and 1 with incongruent data), resulting in 327 correctly completed surveys for the final analysis.

The average age of the students was 22.2 years (SD = 2.3; min = 18; max = 32). Most were women (66.4%), were enrolled in clinical courses (58.7%), were not overweight or obese (68.5%), had family income of <6000 soles (56.9%), had family members with migraine, either first (23.8%) or second degree (40.7%), and had no chronic diseases (70.0%) (Table 1).

**Table 1:** Characteristics of the medical students participating in the study (n = 327).

Characteristics	Total	Migraine		*P*-value
	N (%)	No	Yes	
**Sex**				0.018
Male	110 (33.6)	86 (78.2)	24 (21.8)	
Female	217 (66.4)	142 (65.4)	75 (34.6)	
**Age (years)**^a^	22,25 (2.33)	22.25 (2.16)	22.26 (2.70)	0.952^c^
**Grouped semesters**				0.007
Basic (3–6)	135 (41.3)	83 (61.5)	52 (38.5)	
Clinical (7−12)	192 (58.7)	145 (75.5)	47 (24.5)	
**Family income**^b^				0.867
Less than 6000 soles (PEN)	186 (56.9)	129 (69.4)	57 (30.6)	
More than 6000 soles (PEN)	141 (43.1)	99 (70.2)	42 (29.8)	
**Body mass index**				0.832
Thin/normal	224 (68.5)	157 (70.1)	67 (29.9)	
Overweight/obese	103 (31.5)	71 (68.9)	32 (31.1)	
**Family members with migraine**				0.001
No	116 (35.5)	94 (81.0)	22 (19.0)	
First degree	78 (23.8)	44 (56.4)	34 (43.6)	
Second degree	133 (40.7)	90 (67.7)	43 (32.3)	
**Chronic diseases**				0.540
No	229 (70.0)	162 (70.7)	67 (29.3)	
Yes	98 (30.0)	66 (67.3)	32 (32.7)	
**Insomnia during the pandemic**				<0.001
No	69 (21.1)	60 (87.0)	9 (13.0)	
Yes	258 (78.9)	168 (65.1)	90 (34.9)	
**Exercise during the pandemic**				0.002
No	90 (27.5)	74 (82.2)	16 (17.8)	
Yes	237 (72.5)	154 (65.0)	83 (35.0)	
**Hours of sleep per day**	6.53 (1.22)	6.74 (1.24)	6.06 (1.05)	<0.001^c^
**Hours in front of the computer**	7.7 (2.94)	7.22 (2.59)	8.80 (3.37)	<0.001^c^
**Use of prescription glasses**				0.003
No	125 (38.2)	99 (79.2)	26 (20.8)	
Yes	202 (61.8)	129 (63.9)	73 (36.1)	
**Consumption of food high in fats**				0.165
Once a week	130 (39.8)	85 (65.4)	45 (34.6)	
Two or more times a week	197 (60.2)	143 (72.6)	54 (27.4)	
**Coffee consumption**				0.098
No	68 (20.8)	53 (77.9)	15 (22.1)	
Yes	259 (79.2)	175 (67.6)	84 (32.4)	
**Alcohol consumption**				0.246
No	194 (59.3)	140 (72.2)	54 (27.8)	
Yes	133 (40.7)	88 (66.2)	45 (33.8)	
**Consumption of stimulant beverages**				0.042
No	160 (48.9)	120 (75.0)	40 (25.0)	
Yes	167 (51.1)	108 (64.7)	59 (35.3)	
**Tobacco consumption**				0.107
No	210 (64.2)	140 (66.7)	70 (33.3)	
Yes	117 (35.8)	88 (75.2)	29 (24,8)	
**Do you have or have you had COVID-19?**				0.195
No	173 (52.9)	126 (72.8)	47 (27.2)	
Yes	154 (47.1)	102 (66.2)	52 (33.8)	
**Do you have or have you had a family member with COVID-19?**				0.098
No	121 (37.0)	91 (75.2)	30 (24.8)	
Yes	206 (63.0)	137 (66.5)	69 (33.5)	
**Do you have close family members with risk factors for getting sick with COVID-19 (HBP, diabetes, obesity, older adult, etc.)?**				<0.001
No	124 (37.9)	106 (85.5)	18 (14.5)	
Yes	203 (62.1)	122 (60.1)	81 (39.9)	
**Are you afraid of getting sick with COVID-19?**				0.010
Not afraid	76 (23.2)	62 (81.6)	14 (18.4)	
Little/regular/very afraid	251 (76.8)	166 (66.1)	85 (33.9)	

PEN, Peruvian Currency.

COVID-19, Coronavirus Disease 2019.

HBP, High Blood Pressure.

a Mean and standard deviation.

b 1 USD = 4065 PE N in August 2021.

c Student's t-test.

In addition, 78.9% of the respondents stated that they had had insomnia during the pandemic, 72.5% had exercised during this period, and 61.8% wore prescription glasses. Students slept for an average of 6.5 h (SD = 1.2; min = 3; max = 11) per day and spent 7.7 h (SD = 2.9; min = 1; max = 17) per day on average in front of the computer. Additionally, 47.1% had experienced or had COVID-19, 63% had relatives who also had COVID-19, 62.1% had close relatives with COVID-19 risk factors, and 76.8% were afraid of being infected with COVID-19 (Table 1). Finally, 30.3% (n = 99) of the students had suspected migraine.

In the bivariate analysis, significant differences were found according to suspected migraine in sex, clustered semesters, relatives with migraine, insomnia during the pandemic, exercise during the pandemic, hours of sleep and hours in front of the computer per day, use of prescription glasses, consumption of stimulant drinks, relatives with risk factors for COVID-19, and fear of being infected with COVID-19 (Table 1).

The associations found in the bivariate analysis were found to be significant in the crude model. In the adjusted model, female students were 51% more likely to have migraine than male students. Basic science students were 56% more likely to have migraine than clinical science students. Students with first-degree relatives with migraine were 2.11 times more likely to have migraine than students without relatives having migraine. Students with relatives having COVID-19 risk factors were 85% more likely to have migraines than students without relatives with COVID-19 risk factors. For every hour in front of a computer, the probability of having a migraine episode increased by 6%. For each hour of sleep, the probability of having a migraine episode decreased by 16% (Table 2). No evidence of multicollinearity was found in the adjusted model (VIF <10).

**Table 2:** Multivariate analysis to determine factors associated with migraine in medical students participating in the study (n = 327).

Characteristics	Crude model	*P*-value	Adjusted model	*P*-value
	PR (95% CI)		PR (95% CI)	
Sex				
Male	1		1	
Female	1.58 (1.06–2.36)	0.024	1.51 (1.02–2.22)	**0.035**
**Age (years)**	1.00 (0.93–1.08)	0.956	–	
**Grouped semesters**				
Basic (3–6)	1.57 (1.13–2.18)	0.007	1.56 (1.14–2.13)	**0.005**
Clinical (7−12)	1		1	
**Family income**^a^				
Less than 6000 soles (PEN)	1			
More than 6000 soles (PEN)	0.97 (0.70–1.36)	0.868	–	–
**Body mass index**				
Thin/normal	1			
Overweight/obese	1.04 (0.73–1.47)	0.832	–	–
**Family members with migraine**				
No	1		1	
First degree	2.30 (1.46–3.62)	<0.001	2.11 (1.34–3.31)	**0.001**
Second degree	1.70 (1.09–2.67)	0.020	1.27 (0.82–1.97)	0.275
**Chronic diseases**				
No	1			
Yes	1.12 (0.79–1.58)	0.537	–	–
**Insomnia during the pandemic**				
No	1			
Yes	2.67 (1.42–5.03)	0.002	–	–
**Exercise during the pandemic**				
No	1		1	
Yes	1.97 (1.22–3.18)	0.005	1.36 (0.85–2.16)	0.197
**Hours of sleep per day**	0.72 (0.63–0.82)	<0.001	0.84 (0.72–0.96)	**0.014**
**Hours in front of the computer**	1.12 (1.07–1.20)	<0.001	1.06 (1.01–1.12)	**0.028**
**Use of prescription glasses**				
No	1		1	
Yes	1.74 (1.18–2.56)	0.005	1.40 (0.95–2.07)	0.090
**Consumption of food high in fats**				
Once a week	1			
Two or more times a week	0.79 (0.60–1.10)	0.164	–	–
**Coffee consumption**				
No	1			
Yes	1.47 (0.90–2.40)	0.116	–	–
**Alcohol consumption**				
No	1			
Yes	1.21 (0.90–1.70)	0.245	–	–
**Consumption of stimulant beverages**				
No	1		1	
Yes	1.41 (1.00–2.00)	0.045	1.02 (0.74–1.41)	0.876
**Tobacco consumption**				
No				
Yes	0.74 (0.51–1.08)	0.116	–	–
**Do you have or have you had COVID-19?**				
No	1			
Yes	1.24 (0.89–1.73)	0.196	–	–
**Do you have or have you had a family member with COVID-19?**				
No	1			
Yes	1.35 (0.94–1.95)	0.107	–	–
**Do you have close family members with risk factors for getting sick with COVID-19 (HBP, diabetes, obesity, older adult, etc.)?**				
No	1		1	
Yes	2.75 (1.73–4.35)	<0.001	1.85 (1.15–2.98)	**0.012**
**Are you afraid of getting sick with COVID-19?**				
Not afraid	1		1	
Little/regular/very afraid	1.84 (1.11–3.04)	0.018	0.94 (0.56–1.60)	0.833

PR, prevalence ratio.

95% CI, 95% confidence Interval.

PEN, Peruvian Currency.

COVID-19, Coronavirus Disease 2019.

HBP, High Blood Pressure.

a 1 USD = 4065 PE N in August 2021.

Among students with suspected migraine (n = 99), most reported having two episodes per month (42.4%), an episode length between 1 and 24 h s (72.7%), seeking medical attention (37.4%), or self-medicating (29.3%). Similarly, most students with suspected migraine presented severe functional disability (41.4%) (Table 3).

**Table 3:** Characteristics of migraine episodes (n = 99).

Characteristics	n (%)
**Frequency of migraine episode**	
1 time per year	2 (2.0)
1 time per month	28 (28.3)
2 times a month	42 (42,4)
1–3 times a week	22 (22.2)
4–6 times a week	5 (5.1)
**Duration of migraine episode**	
<1 h	8 (8.1)
1–24 h	72 (72.7)
>24 h	19 (19.2)
**Coping strategies**	
Sought medical attention	37 (37.4)
Self-medicated	29 (29.3)
Slept/Eat	16 (16.1)
Other coping strategy	8 (8.1)
None (endured pain)	9 (9.1)
**Functional disability**	
No disability	24 (24.3)
Mild disability	13 (13.1)
Moderate disability	21 (21.2)
Severe disability	41 (41.4)

## 4 Discussion

Our results show that more than one third of the students had suspected migraine. The following variables were associated with having migraine: female sex, being a basic semester student, history of family members with migraine, family members with COVID-19 risk factors, more hours in front of the computer, and more hours of sleep. Furthermore, most of the students with migraine had severe functional disability.

We found that 30.3% of medical students had suspected migraine. This value is higher when compared with the results of a systematic review with meta-analysis published in 2015, where the combined prevalence of migraine was 16.1%. Although this review included 56 studies with 34,904 undergraduate students, most (62.5%) were medical students, thereby providing a good comparison with our results [21]. In 2018, a similar study conducted in 393 physically healthy students in Italy reported a 26% prevalence of migraine [22]. However, pandemic studies on medical students have described lower prevalence rates. For example, a study in 8783 medical specialty students in China, reported a 6.6% overall prevalence of migraine [23]; a prevalence rate of 12.1% was found in 471 Lebanese students [24]; and the prevalence of migraine and tension headache were 15.3% and 40.3%, respectively, in 352 students in Nepal [25]. These studies used other measurement instruments, which could limit comparability with our results.

Additionally, population studies conducted during the pandemic have found high migraine rates and increased symptoms (longer duration and intensity of the migraine episode) [26]. A worsening of the disease as a consequence of confinement has also been described in patients with migraine [27]. These results suggest, albeit preliminarily, that the pandemic could influence the frequency of migraine in the student population.

Female students were more likely to have migraine than male students. A study reported that women have a higher frequency of migraine episodes, more symptoms (photophobia, phonophobia, nausea, and vomiting), and more migraine-associated disability [28]. Hormonal variations that occur in stages (puberty, menstruation, pregnancy, and menopause) would explain these differences [29].

Students taking basic courses were more likely to have migraine than those taking clinical courses. Some studies have reported similar findings [30], indicating a higher frequency of migraine in first-year medical students; the emotional stress, adaptation, and learning pressures that new students have would explain this result [31]. This higher migraine frequency could be related to more significant academic stress. For example, a higher frequency of migraine was found in Iranian third-year medical students, just when they had an essential basic science examination [32].

Students with family members with risk factors for COVID-19 were more likely to have migraines. The same was observed in students afraid of being infected with COVID-19. Concern for the health of their family members and themselves caused a higher stress level, which is associated with a higher frequency of headaches [33,34]. A multicenter study conducted in Japan in 606 patients with migraine during the first wave of the pandemic reported increased stress levels in 56.8% of the participants [35]. Similarly, an Italian study on 150 patients with migraine conducted during the first wave of the pandemic reported a significant increase in the perceived stress levels associated with the duration and intensity of the migraine episode [36].

Students with first-degree relatives with a history of migraine were twice as likely to have migraine. Migraine is hereditary, with many genetic variants, which, added to environmental factors, give it the denomination of a complex disorder [37]. Several studies have demonstrated the association of family history with migraine, finding differences according to sex [38] and age [39].

Having more hours of sleep was associated with a lower probability of having migraine. A systematic review that evaluated the association of migraine with sleep disorders found that insomnia has a bidirectional relation with migraine, i.e., patients with migraine have a higher risk of developing insomnia. In turn, insomnia is a risk factor for migraine onset, pain intensity, and chronicity of the disease [40]. Similarly, it has been described that sleep disorders can increase anxiety and depression in people with migraine, altering clinical outcomes and responses to treatment [41]. A direct relation has also been described between migraine intensity and psychiatric comorbidities, including its progression to chronicity [42]. In addition, there is abundant evidence showing that anxiety and depression increased considerably during the pandemic [41], so migraine episodes may get worse.

Spending more time in front of the computer was associated with a higher migraine frequency. The confinement caused by the pandemic increased the use of electronic devices, such as computers [43]. Pre-pandemic studies have already described this association; for example, information technology workers in China reported a higher prevalence of tension headaches in those who used computers excessively [44]. Similarly, a study on young French adults reported an increased risk of migraine in those exposed to various screens (computers, tablets, smartphones, and television) [45]. During the pandemic, a study on the Spanish population revealed that electronic devices (primarily computers) increased, on average, by 3.1 h per day, causing symptoms such as headaches in 37% of the participants [43].

In students with migraine, 41.4% had severe functional disability, which is comparable with the results of hospital studies. For example, 37.2% of the patients with migraine attending the neurology service of a South Korean hospital were found to have a severe functional disability [46]. In patients with chronic migraine seen at two clinics in Taiwan, this percentage was 41.3% [47]. We do not know whether these high rates of severe disabilities may be due to the COVID-19 pandemic. To confirm these results, future epidemiological or clinical studies with robust designs and probabilistic sampling are needed.

### 4.1 Limitations

We must mention some limitations. First, the non-probabilistic sampling does not allow the results to represent the population of medical students at the UCSUR; nevertheless, when sampling by quotas (academic semester and sex), the findings would be closer to the students' reality. Second, the self-selection bias could lead to greater participation of students with headaches or migraine. Third, as the study was conducted in only one university, the conclusions cannot be generalized to other Peruvian universities. Fourth, the MS-Q is unable to identify other types of headaches, such as tension headaches, which could explain many of the study's findings, including the high frequency of migraine. Finally, given the study's cross-sectional design, causality between the associated variables cannot be established.

## 5 Conclusion

In conclusion, during the COVID-19 pandemic, medical students presented a high migraine frequency. Given the high level of disability, it is necessary to promote measures aimed at a timely diagnosis and follow-up of affected students. Female students, those from basic courses, with a history of family members with migraine, with family members with risk factors for COVID-19 and those that spent more hours in front of the computer had significantly higher odds of having migraine.

## Author contribution statement

Annabell Zevallos-Vásquez; Kiana Azucena Pajuelo-Salazar: Conceived and designed the experiments; Performed the experiments; Contributed reagents, materials, analysis tools or data; Wrote the paper.

J. Jhonnel Alarco: Conceived and designed the experiments; Analyzed and interpreted the data; Contributed reagents, materials, analysis tools or data; Wrote the paper.

## Data availability statement

Data will be made available on request.

## Additional information

Supplementary content related to this article has been published online at [URL].

## Declaration of competing interest

The authors declare that they have no known competing financial interests or personal relationships that could have appeared to influence the work reported in this paper.
