# Supplementary material for: Migraine, associated factors, and functional disability in medical students at a peruvian university during the COVID-19 pandemic: An analytical cross-sectional study
Source: Heliyon. 2023 Jul 8;9(7):e18108. doi: 10.1016/j.heliyon.2023.e18108 (PMC10362104; doi:10.1016/j.heliyon.2023.e18108)
Supplement: Multimedia component 1 [file mmc1.docx]

**Supplementary Material**

**Frequency of migraine in human medical students at the Universidad Científica del Sur during the COVID-19 pandemic.**

We are Annabell Zevallos Vasquez and Kiana Azucena Pajuelo Salazar, students of Human Medicine at the Universidad Científica del Sur; we are conducting a research entitled: "Frequency of migraine and its associated factors in human medicine students of the Universidad Cientifica del Sur during the COVID-19 pandemic" and we would like to invite students from the 3rd to 10th cycle to participate in this research.

This questionnaire has 40 questions and takes approximately seven minutes to complete. Participation in this research is entirely voluntary; you can choose whether or not you want to participate. If you accept, you will help us realize our project.

The information collected during the research will be kept confidential, and no one other than the researchers will have access to it.

1. Do you agree to participate in the study?
   1. Yes, I accept
   2. I do not accept
2. E-mail: _________________________
3. Are you a student of Human Medicine at the Scientific University of the South?
   1. Yes
   2. No
4. Are you 18 years of age or older?
   1. Yes
   2. No

**Sociodemographic characteristics**

*You will then answer questions regarding your sociodemographic characteristics.*

1. Sex
   1. Man
   2. Woman
2. Age: _______________years
3. Academic cycle
   1. 3rd cycle
   2. 4th cycle
   3. 5th cycle
   4. 6th cycle
   5. 7th cycle
   6. 8th cycle
   7. 9th cycle
   8. 10th cycle
   9. 11th cycle
   10. 12th cycle
4. 11. Approximately how much is your household's monthly income?
   1. Less than 3 thousand soles.
   2. Between 3 thousand to 6 thousand soles.
   3. Between 6 thousand to 9 thousand soles.
   4. More than 9 thousand soles.

**Characteristics of headache**

*Next, answer the questions referring to the characteristics of the headache that occurred during*

*the COVID-19 pandemic.*

1. Do you have frequent or severe headaches?
   1. Yes
   2. No
2. Do your pains usually last more than 4 hours?
   1. Yes
   2. No
3. Do you usually feel nauseous when you have a headache?
   1. Yes
   2. No
4. Are you bothered by light or noise when you have a headache?
   1. Yes
   2. No
5. Does the headache limit your physical or mental activities?
   1. Yes
   2. No
6. How many days did you miss work or school in the last 3 months because of your headache? ____days.
7. How many days did your productivity at work or school decrease by half or less in the last 3 months because of your headache (do not include days you checked in question 1 for missed work or school. If you do not go to school or work, mark zero) ____days.
8. How many days did you not do your household chores in the last 3 months because of your headache? ____days.
9. How many days did your productivity in household chores decrease by half or less in the last 3 months because of your headache (do not include the days you already counted in question 3 for not doing your chores) ____ days.
10. How many days were you unable to participate in family, social and fun activities in the last 3 months because of your headache? ____days.
11. How many days did you have a headache in the last 3 months (if an attack lasted more than one day, count each day) ____ days.
12. On a scale of 0 to 10, how intense were those headaches on average (0: no pain; 10: worst pain imaginable) ____ days.
13. Approximately how frequent are your headache episodes?
    1. 1-3 episodes per week
    2. 4-6 episodes per week
    3. Two episodes per month
    4. One episode per month
    5. Irregular (approximately 1 episode per year)
14. Approximately how long does a headache episode last?
    1. More than 24 hours
    2. Between 1 to 24 hours
    3. Less than 1 hour
15. What coping strategies do you perform when you have a headache episode?
    1. Requested medical assistance
    2. I self-medicate
    3. I sleep
    4. Like
    5. I do nothing (endure the pain)
    6. Another strategy

**Background**

*You will then answer questions about yourself and your family members.*

1. Do you have any family members who have had migraine episodes?
   1. Yes, first-degree relatives
   2. Yes, second-degree relatives
   3. No
2. Do you suffer from any chronic disease? *For example. HT, diabetes, immune disease, obesity, etc.*
   1. Yes
   2. No
3. Do you have or have you had COVID-19 disease?
   1. Yes
   2. No
4. Have you had any family members with COVID-19?
   1. Yes
   2. No
5. Do you have first-degree relatives with risk factors for COVID-19 disease? *For example. HBP, diabetes, obesity, older adult, etc.*
   1. Yes
   2. No
6. Do you suffer, or have you suffered from episodes of insomnia since the pandemic began?
   1. Yes, always
   2. Yes, almost always
   3. Yes, sometimes
   4. No
7. Have you been exercising since the pandemic began?
   1. Yes, always
   2. Yes, almost always
   3. Yes, sometimes
   4. No
8. How many hours, on average, do you sleep per day? _____hours
9. In the last few months, have you perceived that you are overworked?
   1. Yes, for more than 1 month
   2. Yes, for more than 3 months
   3. Yes, for more than 5 months
   4. No
10. Do you wear lenses with measurement?
    1. Yes
    2. No
11. How often do you eat fatty foods?
    1. One day a week
    2. 2-4 days per week
    3. 5-7 days a week
12. Do you drink coffee?
    1. Yes, once a week
    2. Yes, more than 2 times a week
    3. Yes, more than 3 times a week
    4. No
13. Do you drink alcohol?
    1. Yes, once a week
    2. Yes, more than 2 times a week
    3. Yes, more than 3 times a week
    4. No
14. Do you drink stimulant beverages?
    1. Yes, once a week
    2. Yes, more than 2 times a week
    3. Yes, more than 3 times a week
    4. No
15. Do you smoke tobacco?
    1. Yes, once a week
    2. Yes, more than 2 times a week
    3. Yes, more than 3 times a week
    4. No

**Related to computer use**

*You will then answer questions regarding your computer use.*

1. How many hours are you in front of the computer?
   1. 0 to 2 hours
   2. 2 to 4 hours
   3. 4 to 6 hours
   4. More than 6 hours
2. About the previous question, please indicate the number of hours you spend in front of the computer per day (0-24 hours) _____ hours.
